# Potential effect of social support on perceived stress and anxiety in college students during public health crisis: Multiple interactions of gender

**DOI:** 10.1371/journal.pone.0319799

**Published:** 2025-04-10

**Authors:** Taibo He, Chia Ching Tu, Zhi Wang, Danna Hao, Xiaozhou Huang

**Affiliations:** 1 Education Science College, Weinan Normal University, Weinan, China; 2 International College, Krirk University, Bangkok, Thailand; 3 Education Science College, Shaanxi University of Technology, Hanzhong, China; St John’s University, UNITED STATES OF AMERICA

## Abstract

The COVID-19 pandemic has affected college students, leading to increased anxiety and emotional distress. This study investigated how perceived public health crises relate to anxiety levels in college students, and how social support and gender influence this relationship. Data from 3,165 college students from six universities in Shaanxi Province, China, were collected and analyzed by using AMOS and SPSS PROCESS 4.0. Results showed that perceived COVID-19 risk significantly impacted anxiety levels, and social support moderated this relationship. Gender also had multiple interaction effects with social support and perceived pandemic risk on anxiety. Overall, the study confirms that COVID-19 quarantine and perceived risk increase stress and anxiety in college students, with social support playing a buffering role, albeit with variations based on gender.

## Introduction

The global public health crisis caused by the COVID-19 pandemic has led to hundreds of thousands of infections and a significant loss of life. As of January 17, 2022, the World Health Organization reported approximately 326 million confirmed cases and over 5.5 million deaths worldwide. The rapid spread and scale of the pandemic have posed serious health challenges, straining healthcare systems and resources in numerous countries. The direct risks to individuals’ lives and health are evident, with elevated levels of anxiety and emotional distress being widely observed. Isolation has proven to be one of the most effective measures to prevent the spread of COVID-19. However, prolonged isolation, semi-isolation, and social controls have profoundly disrupted normal educational activities, particularly affecting students across all levels of education. Among these, university students have faced unprecedented mental pressures [1–4]. While the impact of the pandemic on the lives and studies of university students is gradually diminishing, the prolonged presence of the virus has become normalized, and the resultant psychological pressures should not be underestimated [5–7]. Thus, research on the mental well-being of university students during the pandemic remains critically important.

Studies have revealed that college students exhibited higher overall anxiety levels during the COVID-19 period compared to the general population [8], with an anxiety incidence rate of 26.6% [9]. Anxiety, depression, stress, and sleep disorders were common psychological consequences of COVID-19 [10]. Anxiety may lead to the escalation of mental health complications, resulting in poor academic performance, academic failure, and even suicide attempts [11].

Therefore, this study examined the potential effect of perceived COVID-19 risk on college students. Additionally, exploring personal and social factors that could buffer such effects may provide valuable practical insights. College students, who are transitioning from adolescence to adulthood, are typically passionate and ambitious. However, due to their developing identities and limited self-regulation, they often grapple with inner psychological conflicts and confusion when confronted with perceived COVID-19 risks. Moreover, they may be more vulnerable to negative emotions, such as anxiety and depression [2,12–14].

The stress-buffering hypothesis of perceived social support suggests that perceived social support can mitigate the psychological trauma caused by negative events [15]. Therefore, we hypothesize that when college students received greater social support during school lockdowns, their anxiety levels were reduced. However, studies have not yet confirmed gender differences in social support. Nevertheless, previous research has shown that many psychiatric disorders affect females and males differently, with incidence rates of certain disorders varying by gender. For example, females are more likely to be diagnosed with posttraumatic stress disorder and major depression than males [16–18]. Gender-specific differences have also been observed in responses to psychological stress during the COVID-19 pandemic [19,20]. Thus, investigating the potential effect of perceived pandemic risk on the mental health of college students, especially with regard to gender-specific differences, is warranted.

Additionally, psychopathological models that focus on variables influencing individuals’ adaptation to environmental stress can be applied to analyze the role of social support in coping with COVID-19 lockdowns [21].

Therefore, this study examined the possible effect of perceived COVID-19 risk on college students ’anxiety levels in the context of university closures, with a focus on (1) whether there is a potential association between perceived COVID-19 risk and college students’ anxiety levels during quarantine. (2) whether social support potentially moderated the association between perceived COVID-19 risk and anxiety among college students. (3) whether gender-specific differences in social support moderated the association between perceived COVID-19 risk and anxiety among college students during the COVID-19 pandemic.

## Literature review

Selye’s [22] systemic theory portrays stress as a pattern of nonspecific responses or changes in the body induced by any imposing demands. This definition implies that critical life events, such as the COVID-19 pandemic, are likely to test individuals’ mental resources and ultimately trigger coping strategies. The present study indicates that perceived COVID-19 risk is one such factor that predicts poor emotional outcomes. Perceived COVID-19 risk refers to the perception of risk and the stress experienced during COVID-19 outbreaks [20]. One study indicated that individuals’ perceived stress increases when they are faced with external pressure and limited resources [23]. Another study suggested that perceived COVID-19 risk was a major source of daily stress among college students during the pandemic, and the predominant source of stress was perceived pandemic risk [24]. Approximately 35% of respondents experienced varying degrees of psychological stress due to the COVID-19 pandemic [25]. The COVID-19 pandemic caused increased anxiety due to the stress triggered by perceived pandemic risk and other factors [26]. Whether college students’ perceived pandemic risk affected their anxiety levels merits investigation. Studies must analyze the effects of isolation during a pandemic. Therefore, this study proposes the following hypothesis:

*Hypothesis 1*: Perceived COVID-19 risk significantly affected the anxiety levels of college students during pandemic-imposed isolation.

Social support has been described as “support that individuals gain through their social connections with other individuals, groups, or larger communities” [27]. Evidence has indicated that social support predicts mental health outcomes. Perceived social support under stress helps protect against poor mental health outcomes [28–31]. College students with various levels of social support have significant differences in anxiety levels, and social support is negatively associated with anxiety [31,32]. Social support can buffer the effects of anxiety generated by perceived pandemic risk and can improve an individual’s physical and mental health [21]. Social support refers to the maintenance of identity and the emotional support gained through social relationships [33]. A relevant study suggested that social support can reduce the negative effects of stress on psychological anxiety [34]. Perceived pandemic risk is a negative stressor for college students; therefore, this study proposes the following hypothesis:

*Hypothesis 2*: College students’ perceived social support significantly moderated the relationship between their perceived COVID-19 risk and anxiety levels.

Scholars have long emphasized gender-specific differences in the mental health challenges of college students. Many studies have demonstrated that female students tend to experience higher stress and anxiety levels [35–38] and exhibit more visible psychological symptoms [39–41]. Some researchers have indicated that male students are more susceptible to mental illness than female students [42,43].

Male and female college students differ with respect to perceived COVID-19 risk, and female students experienced greater vulnerability after the emergence of COVID-19 [44]. One study indicated that the risk-related stress caused by the COVID-19 pandemic is not neutral but influenced by gender-specific differences [45]. females tend to be more sensitive to stress than males, and their own vulnerability in the face of stress often leads to psychological distress. A study by scholars in Spain found that compared with male students, female students’ mental health was more severely affected by the COVID-19 pandemic, as evidenced by them having higher levels of anxiety, emotional problems, and physical discomfort [46]. Depression, anxiety, and suicidal ideation were also more common in girls in a sample of Chinese and Jordanian students [47]. A study of college students indicated that COVID-19 had a more significant and negative effect on academic performance, stress, and mental health in female students than in male students [20]. College students with higher levels of social support have lower levels of stress. College students’ perceived levels and sources of social support reveal clear gender differences. One study indicated that female students reported higher levels of social support and stress than did male students [48]. A study of the college student population revealed a significant disparity between males and females in access to social support [49]. Therefore, this study proposes the following hypothesis:

*Hypothesis 3*: The interaction between gender and social support can moderate anxiety levels in college students generated by the stress of perceived COVID-19 risk.

## Methods

**Fig 1:**
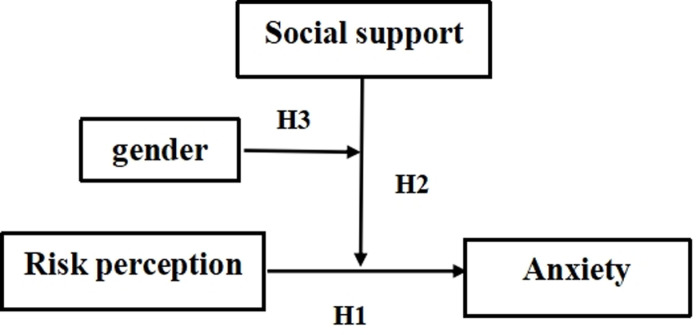
Research structure.

In the present study, a model (Fig 1) was constructed in accordance with a literature review, Selye’s systemic theory, and the stress-buffering hypothesis of social support. Gender exerted multiple interaction effects on the relationship between social support and anxiety levels in college students during COVID-19 isolation.

### Participants and procedures

This study was approved by the Scientific Research Ethics Committee of Weinan Normal University. Given the phased and regional outbreaks of the COVID-19 pandemic, and considering that random sampling requires more time, planning, and funding, purposive sampling was chosen for this study due to its advantages of higher participant cooperation and questionnaire return rates. The study selected six universities in Shaanxi Province that were under campus lockdown due to the pandemic. The sampled students majored in fields such as engineering, medicine, science, literature, law, business, and education. With participants’ consent, mental health counseling teachers from the six universities organized the survey, which included students from all academic years, ranging from freshmen to seniors. A stratified sampling method was used for the survey, with two classes of students in each grade, in the various majors selected to participate. The study participants were recruited on a class basis, with voluntary participation; those who were not willing to participate could choose not to complete the questionnaire. Which began on 6 January 2022 and concluded on 15 January 2022, through the professional survey platform Questionnaire Star (https://www.sojump.com). the questionnaire consisted of 49 items, including 4 demographic questions (age, gender, school year, and family economic status). The online questionnaire was distributed to students (M age =  19.58, SD age =  1.28, age range =  18 to 24 years; males =  1,890, females =  1,275; see Table 1) affected by the COVID-19 pandemic, all of whom had been in full lockdown for approximately 12 days. A total of 3,400 questionnaires were distributed, and after excluding responses with missing answers and repetitive patterns, 3,165 valid responses were obtained, resulting in a questionnaire effectiveness rate of 92.8%. The study consisted of 41 items and a sample size of 3,165, which aligns with the standard requirement for SEM research, where the sample size is generally recommended to be at least 10 times the number of items [50].

**Table 1:** Demographic descriptive statistics (*N* =  3,165).

Basic information	Types	Frequency	Proportion (%)
Gender	Male	1,890	59.7
	Female	1,275	40.3
Region	City proper	636	20.1
	Suburb	77	2.4
	Rural area	2,452	77.5
School year	Freshman	1,337	42.2
	Sophomore	1,311	41.4
	Junior	416	13.1
	Senior	101	3.2
Family economic status	Richer	29	0.9
	Rich	21	0.7
	Average	1,768	55.9
	Low-income	1,090	34.4
	Extremely poor	257	8.1

### Research tools

#### Social support.

The Chinese version of the Multidimensional Scale of Perceived Social Support (MSPSS-C) was revised by Chinese scholar Jiang [51] based on the scale created by Zimet et al. [52]. This scale analyzes three dimensions of social support: the support of family (e.g., “When needed, I can seek emotional help and support from family”), the support of friends (e.g., “In times of difficulty, I can count on my friends”), and the support of others (e.g., “When I have difficulties, my teacher is a true source of comfort”). The scale comprised 12 items, with 4 items in each dimension. This study scored the responses based on a 5-point Likert-type scale, ranging from 1 (*complete nonconformity*) to 5 (*complete conformity*). A higher score indicated more social support. The Cronbach’s α values for the three dimensions of the scale were 0.92, 0.87, and 0.87.

### Anxiety

The Zung Self-Rating Anxiety Scale (SAS) [53] is composed of 20 questions divided into four dimensions of symptom manifestations of experiencing anxiety. It includes 5 questions each for cognitive symptoms (e.g., “I feel worried for no reason”), sensory motor tension (e.g., “I feel like I will faint” and “I feel my heart beating faster”); and motor and central nervous system symptoms (e.g., “I experience restless sleep and insomnia”) [54]. This study employed a Likert-type scale to score the responses, ranging from 1 (complete nonconformity) to (complete conformity) points. A higher score indicated more severe anxiety. Studies have indicated that this widely used scale has good reliability and validity [32]. The Cronbach’s α values for the four dimensions of the scale were 0.92, 0.92, 0.87, and 0.83.

### COVID-19 risk perception

The Perceived Risk of COVID-19 Pandemic Scale, compiled by Chinese scholars Xi Juzhe and She Zhuang [23], is used to examine three dimensions. The dimensions are emotional feelings (e.g., “I am worried about becoming infected with the new coronavirus”), cognitive judgment (e.g., “I am sure I will not become infected with the new coronavirus”), and catastrophe characterizations (e.g., “I often think about what will happen if I’m infected with COVID-19”). Responses were rated on a 5-point Likert-type scale ranging from 1 (*completely disagree*) to 5 (*completely agree*). A higher score indicated a higher level of COVID-19-related stress. The Cronbach’s α values for the three dimensions of the scale were 0.89, 0.92, and 0.85.

### Data analysis

Data were analyzed using AMOS version 26.0 (IBM Corporation), SPSS PROCESS 4.0 [55]. A reasonable measurement model was tested by using the confirmatory factor analysis (CFA) [56–58], and model parameters and fit indices obtained from the Asymptotically distribation-free were taken as statistical indicators to confirm the collocation degree between 3,165 students’ data and the measurement mode. Following the recoding of reverse-scored items, two reverse-scored items from the Social Support scale and one from the Risk Perception scale and four from the Anxiety scale were removed due to low factor loadings. The 34 valid items retained in the CFA model demonstrated satisfactory factor loadings (all >  0.50) [50]. The Cronbach’s α values for the Risk Perception,Social Support, and Anxiety scales were 0.924, 0.928, and 0.953.Cronbach’s α of the three scales ≥ 0.7 indicates good reliability, CR (Composite reliability) ≥ 0.7 suggests that the scale has excellent composite reliability, and AVE (Average variance extracted) ≥ .50 indicates that the scale has good convergent validity [59,60].values meet the criteria (Table 2), The sample size meets the requirement of a 99% confidence level, with a sampling error set within a margin of ± 5%.To validate the regression assumptions, the Durbin-Watson test was conducted. The result showed a Durbin-Watson value of 1.799, which falls within the 1.5 < Durbin-Watson <  2.5 range, indicating residual independence and meeting the independence assumption for regression analysis [61,62].

**Table 2:** Cronbach’s α, composite reliability (CR), and average variance extracted (AVE) values.

	Cronbach’s α	CR	AVE
**Risk Perception (RP)**	0.924	0.928	0.724
**Anxiety (AN)**	0.953	0.955	0.705
**Social Support (SS)**	0.928	0.936	0.669
**Standard**	≥0.7	≥0.7	≥0.5

*N* =  3,165.

The CFA model met the criteria for preliminary model fit, including no negative significant error variances, correlations ranging from -0.11 to 0.36, and factor loadings between 0.61 and 0.94. Thus, the reliable results of the CFA model reflect the measurement validity of the three scales. First, the model fit indices for the CFA were acceptable (χ² =  1981.381, df =  406, χ²/df =  4.88, RMSEA =  0.035, SRMR =  0.038, GFI =  0.961, CFI =  0.985, AGFI =  0.949, NFI =  0.981, TLI =  0.982), All of these shall meet the standards[56,63–68]. Furthermore, to avoid the problem of multicollinearity, we centralized the data. The reliability and validity indexes of the three latent variables and the measurement model fit index are shown in Table 3. COVID-19 R.P has a χ^2^/d > 5. In Structural Equation Modeling (SEM), the reasonable threshold for the chi-square to degrees of freedom ratio (CMIN/DF) often varies depending on the complexity of the model and the sample size. Typically, a CMIN/DF below 5 is considered an acceptable standard for model fit (citation), but in cases where the model is more complex, higher thresholds may sometimes be acceptable, extending up to 8 [58,69]. CFI, NFI and TLI are less than 0.9, but greater than 0.8. However, considering the complexity of the model and the large number of samples, CFI, TLI and NFI values are between 0.8-0.9, which also belong to the acceptable range [59,68].

**Table 3:** Measurement model fit index.

Model	*χ* ^*2*^	*χ* ^*2*^ */df*	CFI	GFI	NFI	RMSEA	SRMR	TLI
Social support	163.518	4.542	0.945	0.950	0.931	0.033	0.022	0.916
COVID-19 R.P	109.256	7.964	0.918	0.960	0.912	0.056	0.049	0.812
Anxiety	345.129	3.196	0.887	0.902	0.847	0.030	0.077	0.837
Standard		≤0.8	≥0.8	≥0.8	≥0.8	≤0.08	≤0.08	≥0.8

*N* =  3,165.

### Descriptive statistics

This study implemented the SPSS 26 PROCESS model 3 to analyze the interaction effects and employed hierarchical regression analysis to explore the influence of perceived pandemic risk on the anxiety levels of college students in COVID-19 isolation and lockdown (Table 4). Furthermore, this study analyzed the moderating effect of social support on perceived COVID-19 risk and anxiety and the interaction moderating effect of gender. The second- and third-order interaction moderating hypothesis models were analyzed and tested for each variable.

**Table 4:** Correlations and descriptive information of variables.

Variable	MEAN	SD	AN	RP	SS
**Age**	19.58	1.280			
**Anxiety(AN)**	2.107	0.678	**0.84**		
**Risk Perception(RP)**	1.859	0.688	0.371^***^	**0.851**	
**Social Support(SS)**	3.753	0.718	-0.279^***^	-0.11^***^	**0.818**

*N* =  3,165;

***p < 0.001.

*The bold diagonal elements are the square roots of each AVE; construct correlations are shown off-diagonal.

## Results

This study included the variables of age, location, and family economic status in Model 1 to examine the effect of perceived COVID-19 risk on college students’ anxiety levels under COVID-19 isolation. The results revealed that perceived COVID-19 risk significantly influenced students’ anxiety levels (*β*= 0.791, *p* < .001), and differences in family economic status also significantly affected anxiety levels generated by perceived COVID-19 risk (*p* <  0.01).

### Interaction test of social support

**Fig 2:**
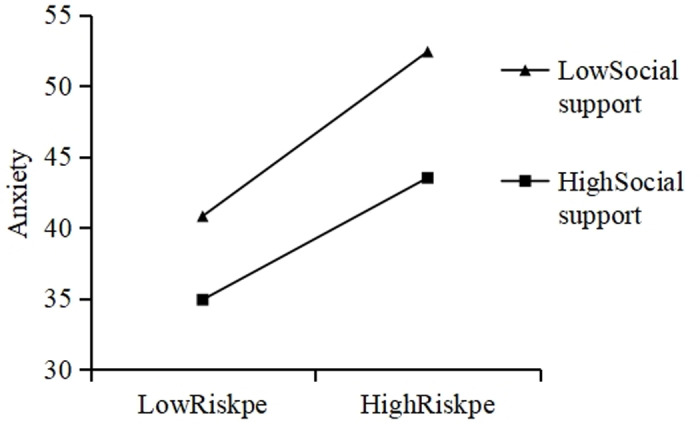
Interaction diagram.

The second-order interaction term “perceived COVID-19 risk × social support” was added to Model 2 to explore the role of social support between perceived COVID-19 risk and anxiety in college students during COVID-19 isolation. The level of social support significantly regulated perceived COVID-19 risk and anxiety (*p* <  0.001) (Fig 2). High levels of social support significantly reduced anxiety caused by perceived COVID-19 risk.

### Analysis of interaction effects

The third-order interaction term “perceived COVID-19 risk × social support × gender” was added to Model 3 to explore the various roles of social support in the relationship between perceived COVID-19 risk and anxiety in college students during COVID-19 isolation. The effects of social support and perceived pandemic risk were significant (*p* <  0.001) (Table 5).

**Table 5:** Hierarchical regression analysis of the influence of gender and social support on anxiety.

Variable	Anxiety					
**Model 1**		**Model 2**		**Model 3**	
	*β*	*t*	*β*	*t*	*β*	*t*
Age	-.258	-.1.389	-.300	-1.675	-.325	-1.801
Economic status of family	1.051	3.065^**^	0.892	2.695^**^	0.923	2.742^**^
**Independent variable**						
Risk perception	0.791	22.650^***^	0.783	23.010^***^	0.816	7.895^***^
Social support			-0.384	-14.876^***^	-0.357	-4.576^***^
Gender					0.112	0.233
**Two-way interaction term**						
Risk perception of COVID-19 * social support			0.012	-3.723^***^	-0.048	-4.628^***^
Risk perception of COVID -19 * gender					-0.012	-0.157
Social support*gender					-0.014	-0.251
**Three-way interaction term**						
Risk perception of COVID-19 * social support*gender					0.029	3.609^***^
*R*	0.377		0.447		0.449	
*R* ^*2*^	0.142		0.202		0.205	
*F*	174.670^***^		159.697^***^		90.473^***^	

*N* = 3,165; * *p* < 0.05,

**p < 0.01,

***p < 0.001; The female code was 0 and the male code was 1, with girls as the reference group.

### Effect of gender on anxiety levels under the interaction of social support and perceived risk

This study included the interaction term of social support and perceived stress in Model 2 to examine the effect of such interaction on anxiety levels by gender. The results indicated that the interaction between social support and perceived stress significantly and negatively predicted anxiety levels in male college students (*Bsimple* =  − 0.019, *p* <  0.001), whereas the interaction effect was nonsignificant in female college students (*Bsimple* =  0.009, *p* =  0.204) (Table 6).

**Table 6:** Multiple interaction tests of the effects of gender on perceived pandemic risk and social support.

Gender	Effect	*F*	*df*1	*df*2
1(Male)	-0.019^***^	24.296	1	3157
2(Female)	0.009	1.613	1	3157

*N*= 3,165;

***p<0.001.

**Fig 3:**
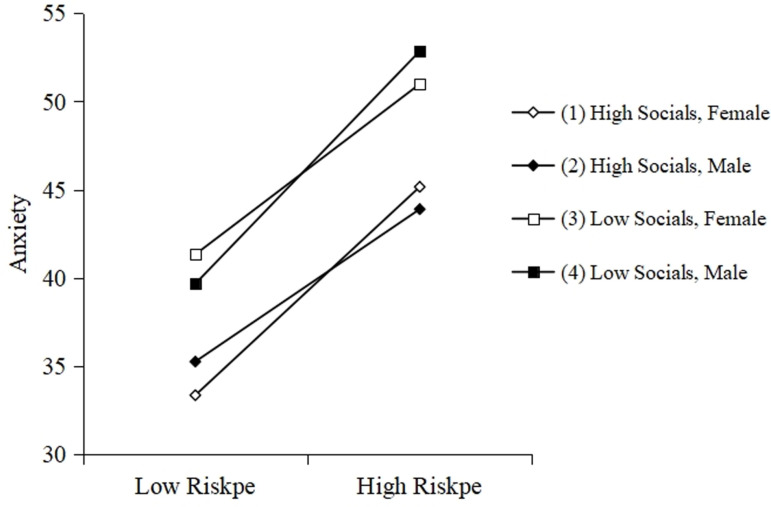
Third-order interaction effects of gender and social support on anxiety levels under perceived epidemic stress.

To further analyze the moderating pattern of the third-order interaction of stress perception ×  social support ×  gender, stress perception and social support were grouped into high and low subgroups according to M ± 1SD, and gender was coded as 1 (males) and 2 (females) for the simple slope test. The results are shown in Fig 3 and Table 7. For the high social support subgroup, the positive effect of epidemic stress perception on anxiety level was significant for male college students (Bsimple = 0.636, *p* < 0.001). For female college students, the positive effect of epidemic stress perception on anxiety level was also significant (Bsimple = 0.869, *p* < 0.001) and may be even stronger. For the low social support subgroup, the positive effect of perceived epidemic stress on anxiety levels was significant for both male college students (Bsimple = 0.97, *p* < 0.001) and for female college students (Bsimple = 0.71, *p* < 0.001) and possibly weaker.

**Table 7:** Slope difference tests.

	Slope 1	Slope 2	Slope 3	Slope 4
	Socials = 8.894	Socials = 8.894	Socials = -8.894	Socials = -8.894
	gender = 2	gender = 1	gender = 2	gender = 1
Bsimple	0.869	0.636	0.710	0.970
*t*-value	9.262	13.242	8.544	16.363
*p*-value	0.000^***^	0.000^***^	0.000^***^	0.000^***^

*N*=3,165; *p<0.05, ** *p*<0.01,

***p<0.001.

In order to compare the four simple slopes, we tested the difference in the slopes of the effect of perceived epidemic stress on anxiety for different combinations of high and low levels of the two moderating variables, as suggested by Dawson and Richter (2006) (Table 8). The results showed that the slope of “female college students + high social support” was significantly larger than that of “male college students + high social support”, indicating that perceived epidemic stress caused more anxiety among female college students when they were also high in social support. The slope of “female college students + low social support” is significantly smaller than that of “male college students + low social support,” which indicates that the perceived stress of the epidemic will bring more anxiety to male college students when they have the same low social support. The slope of “male college students + high social support” is significantly smaller than that of “male college students + low social support,” suggesting that social support can buffer the positive effect of perceived epidemic stress on anxiety for male college students.

**Table 8:** Comparison of the single slopes of the third-order interaction effects.

Pair of slopes	Slope difference	t-value	95% Confidence interval
(1) and (2)	0.233^*^	2.232	(0.028, 0.438)
(1) and (3)	0.158	1.247	(-0.091, 0.407)
(1) and (4)	-0.101	-0.907	(-0.320, 0.118)
(2) and (3)	-0.075	-0.772	(-0.265, 0.115)
(2) and (4)	-0.334^***^	-4.853	(-0.469, -0.199)
(3) and (4)	-0.260^*^	-2.558	(-0.459, -0.061)

*N*=3165;

*p<0.05,

***p<0.001.

## Discussion

### Relationship between perceived COVID-19 risk and college students’ anxiety levels during isolation

The quarantine measures to prevent the spread of the COVID-19 pandemic drastically limited social interactions and psychologically challenged college students who had just transitioned to life on college campuses. The hierarchical regression analysis results supported Hypothesis 1. In accordance with the scores of the SAS, the overall anxiety level during school closure and isolation was approximately 29.5%, higher than the level (26.6%) under pandemic conditions [9]. The results of this study demonstrated that the overall anxiety level of college students during COVID-19 university closures was higher than that in the general public [8,9,25,28,32]. College students are in their youth, a period of enthusiastic struggle. However, to prevent the spread of the pandemic, the Chinese government implemented measures such as border closures and quarantine. During the scheduled winter vacation, COVID-19 interrupted the lives and study schedules of college students, who were forced to remain in their dormitories, unable to reunite with relatives and friends. The normal pressures of school, life, and emotions combined with perceived pandemic risk led to significantly higher levels of anxiety during isolation. The results revealed that perceived COVID-19 risk positively predicted anxiety levels, consistent with the results of relevant studies. Therefore, the psychological status of college students during COVID-19 isolation merits in-depth investigation.

### Correlation analysis of the effects of social support on anxiety and perceived COVID-19 risk

This study demonstrates that social support moderated the relationship between anxiety and perceived COVID-19 risk (*β*= − 0.356, *p* < .001), which is consistent with the results of previous studies [25–28,31]. Therefore, the results verify Hypothesis 2. Social support theory posits that support from family, friends, and other relationships enables individuals to better cope with greater stress. Social support can alleviate both stress and anxiety and thus exerts multiple interaction effects. Therefore, when implementing COVID-19 quarantine measures, universities should provide increased social support services and enhance students’ awareness of COVID-19 risks.

### Interactions between gender and social support and their effect on the relationship between perceived pandemic risk and anxiety

The results of this study reveal that the male college students who received high levels of social support had lower levels of anxiety than did male students who received low levels of social support. As perceived pandemic risk increased, the male college students who received high levels of social support maintained lower levels of anxiety than did their male counterparts with low levels of social support. However, the results indicate that the anxiety levels of female college students generated by perceived pandemic risk did not significantly change regardless of the level of social support received.

The present study demonstrates that the effect of perceived COVID-19 risk on the anxiety levels of college students in isolation varied by gender. Consistent with the results of previous studies, our results indicate significant differences in anxiety levels caused by perceived pandemic risk in male and female students with different levels of social support [19,20,47,66]. This result validates Hypothesis 3.

The aforementioned results suggest that male and female students differ with respect to personality type; female students prefer to communicate and share with others more than their male counterparts [35]. In adverse situations, female students can obtain comfort and support from others [44,46], which can alleviate lockdown-related anxiety. females in China regularly seek and receive support from family and friends during both ordinary and extraordinary times. However, traditional patriarchal education in China emphasizes bravery and responsibility in males [70,71], neglecting the aspects of care and affection. Because males tend to share their worries with others less often, they may experience more intense anxiety when they face adversity, such as during COVID-19 lockdowns. In such situations, male university students with greater social support are better able to cope with the anxiety caused by perceived COVID-19 risk than those with lower social support. This finding may help school administrators devise an effective method of alleviating anxiety in university students during pandemic lockdowns.

The results of this study reveal that social support and perceived pandemic risk affected anxiety levels in university students. However, the moderating effects of social support on anxiety levels differed by gender. This finding corroborates the stress-buffering hypothesis of social support [18] and provides a reference for those designing psychological health interventions for university students.

In this study, the effect size of Risk Perception on Anxiety was *β* =  0.371, Social Support on Anxiety was *β* =  -0.279, and Social Support on Risk Perception was *β* =  -0.011. According to the standard proposed by Kenny, where *β* ≥  0.25 is considered a medium effect, the effect size of Social Support on Risk Perception (*β* =  -0.011) constitutes a weak effect [72]. However, based on Rosenthal and Rosnow’s recommendation and given the large sample size in this study (*N* =  3165), along with the complexity of psychological phenomena among university students, the effect size of Social Support on Risk Perception (*β* =  -0.011) remains interpretable and meaningful in this context [73,74].

## Conclusion

In accordance with Selye’s [22] systemic theory and the stress-buffering hypothesis of social support [15], this study constructed a model of the moderating interaction between gender and social support that affected college students’ anxiety levels under perceived COVID-19 risk. The results of this study enrich knowledge of college students’ mental health during public health crises and can guide college students to overcome stressful events such as lockdowns.

### Recommendations

This study proposes the following recommendations. First, school administrators should work to reduce the perception of risk among university students during public health crises. In addition to following pandemic prevention policies, college students should understand the international pandemic situation and the diminished effects of the evolving virus. Universities should not spread the fear of infection among college students, but rather encourage positive strategies to live with the virus. Physical and mental health are the most effective tools of resistance. Second, universities should improve social support services for college students, particularly male students. Furthermore, they should encourage interaction between male and female college students, which can allow male students to learn how to seek support from others and allow female students to gain courage from male students. Encouragement during times of adversity can enable students to overcome difficulties together.

### Limitations and future directions

Since this study utilized a cross-sectional design, our findings may be limited to the psychological responses of the sample at a specific point in time and under particular environmental conditions, identifying only potential influences. Therefore, further longitudinal research is necessary to explore how the model may change over time. Secondly, as the sample for the questionnaire was selected using purposive sampling, generalizing the findings to the broader student population requires more extensive and comprehensive studies. Additionally, this study employed quantitative methods to examine university students affected by campus lockdowns during the COVID-19 pandemic. Some model fit indices (e.g., GFI, TLI) did not meet the most ideal standards, which may be attributed to factors such as the sample size, the complexity of the model, and the limited number of variables. In future research, alternative qualitative methods could be applied to further validate our findings. Finally, as this study was conducted within a mixed-gender sample, the findings may not be directly applicable to homogenous groups. Researchers could test this model among more homogenous student groups to evaluate its generalizability.

## Supporting information

S1 File Renamed_a9368. (SAV)
